# Estimating genetic diversity across the neutral genome with the use of dense marker maps

**DOI:** 10.1186/1297-9686-42-12

**Published:** 2010-05-10

**Authors:** Krista A Engelsma, Mario PL Calus, Piter Bijma, Jack J Windig

**Affiliations:** 1 Wageningen UR Livestock Research, Animal Breeding and Genomics Centre, PO Box 65, 8200 AB Lelystad, The Netherlands; 2 Wageningen University, Animal Breeding and Genomics Centre, PO Box 338, 6700 AH Wageningen, The Netherlands; 3 Centre for Genetic Resources, The Netherlands (CGN), PO Box 65, 8200 AB Lelystad, The Netherlands

## Abstract

**Background:**

With the advent of high throughput DNA typing, dense marker maps have become available to investigate genetic diversity on specific regions of the genome. The aim of this paper was to compare two marker based estimates of the genetic diversity in specific genomic regions lying in between markers: IBD-based genetic diversity and heterozygosity.

**Methods:**

A computer simulated population was set up with individuals containing a single 1-Morgan chromosome and 1665 SNP markers and from this one, an additional population was produced with a lower marker density i.e. 166 SNP markers. For each marker interval based on adjacent markers, the genetic diversity was estimated either by IBD probabilities or heterozygosity. Estimates were compared to each other and to the true genetic diversity. The latter was calculated for a marker in the middle of each marker interval that was not used to estimate genetic diversity.

**Results:**

The simulated population had an average minor allele frequency of 0.28 and an LD (r^2^) of 0.26, comparable to those of real livestock populations. Genetic diversities estimated by IBD probabilities and by heterozygosity were positively correlated, and correlations with the true genetic diversity were quite similar for the simulated population with a high marker density, both for specific regions (r = 0.19-0.20) and large regions (r = 0.61-0.64) over the genome. For the population with a lower marker density, the correlation with the true genetic diversity turned out to be higher for the IBD-based genetic diversity.

**Conclusions:**

Genetic diversities of ungenotyped regions of the genome (i.e. between markers) estimated by IBD-based methods and heterozygosity give similar results for the simulated population with a high marker density. However, for a population with a lower marker density, the IBD-based method gives a better prediction, since variation and recombination between markers are missed with heterozygosity.

## Background

Conservation of genetic diversity in livestock is of vital importance to cope with changing environments and human demands [1]. Intensive livestock production systems have limited the number of breeds and lines used, and many native breeds have become rare or extinct, causing a loss of genetic diversity. To conserve biodiversity and ensure its sustainable use, efforts are being made world-wide [2], for example in the form of genetic diversity conservation via gene banks or by maintaining genetic diversity in breeding populations. Determining and evaluating genetic diversity present within livestock breeds are crucial to make the right conservation decisions and to efficiently use resources available for conservation.

To evaluate genetic diversity in livestock populations, several methods have been developed [3]. These methods are based on pedigree information, or on molecular data when pedigree information is not available. During the last decade, availability and use of molecular information have increased, and numerous types of markers have become available to evaluate genetic diversity. Microsatellites have been widely used for conservation purposes, but are gradually being replaced by SNP markers which are available in large numbers across the entire genome. These dense marker maps enable us to evaluate genetic diversity more precisely and to obtain information on the genetic diversity separately for each specific segment of the genome.

Basically, there are two approaches to evaluate genetic diversity. In molecular and population genetics, heterozygosity of markers is the most widely used genetic diversity parameter [4]. In quantitative genetics and animal breeding, additive genetic variance of traits estimated with the help of pedigrees is generally used to evaluate genetic diversity [5]. To determine additive variance with markers, the probability that two alleles are identical by descent (IBD), i.e. originate from the same ancestral genome, is estimated [6]. The probability of IBD is closely related to the relationship coefficient (*r*) calculated from pedigrees for the estimation of additive variance. Although theoretically both approaches should give similar results, in practice they are weakly correlated [7,8]. As dense marker maps have become available, it is possible to estimate additive genetic effects of markers and this is routinely used in, for example, QTL-detection [9] and genomic selection [10,11].

A crucial difference between heterozygosity on the one hand and IBD probabilities and *r *on the other hand is that the latter depend on a base population. Markers can be alike in state (AIS) but not IBD if they originate from different ancestors in the base population. With heterozygosity this distinction is not made. For example, in the case of QTL detection, IBD probabilities are used because they better predict whether two chromosome intervals carry the same QTL. The reason is that if an individual carries markers at two loci around an interval that are both AIS, but not IBD (i.e. originate from different ancestors), it is less likely that the interval between the markers is completely AIS and carries the same QTL. However, if both markers are IBD the interval will also be IBD (and AIS), unless a double recombination has occurred in the interval.

Both heterozygosity and IBD probabilities can be used to estimate genetic diversity in specific regions of the genome, in which it may deviate from the average diversity calculated over the whole genome. Heterozygosity and IBD probabilities as genetic diversity measures may also deviate from each other. It is unclear how substantial the difference is between the two approaches and whether it varies over the genome. These local differences may be averaged out if the average diversity is calculated over the whole genome. However, both approaches can be used to estimate the genetic diversity for sequences lying in between genetic markers. Because IBD probabilities are used specifically to predict the presence of QTL between markers one may expect that IBD probabilities better predict genetic variation between markers. Whether this is a substantial difference is not clear.

The aim of this paper was to compare two different estimates of the genetic diversity of a region lying in between markers over the genome i.e. IBD probabilities between marker haplotypes and heterozygosity. Towards this aim, we generated genetic diversity over a genome by computer simulation of two populations each with a different marker density. IBD-based genetic diversity and heterozygosity were compared for the average diversity of regions in the genome containing several marker intervals, and for the genetic diversity at each marker interval. To evaluate how well these estimates predict the genetic diversity over the genome, both were compared to the true genetic diversity.

## Methods

A population was computer simulated with neutral SNP markers across the genome. Next, for each locus in the genome, the genetic diversity was estimated in three ways: (1) based on IBD probabilities with flanking markers; (2) based on expected heterozygosity with flanking markers; (3) the true expected heterozygosity of the marker itself. For (1) and (2), the marker at the locus itself was assumed to be unknown. In this way the predicted diversities (1) and (2) could be compared with true genetic diversity (3).

### Simulated population

Simulations were aimed at generating a population with a neutral genetic diversity varying over the genome. We avoided selection as this may cause specific patterns in genetic diversity (e.g. selective sweeps). Variation in diversity in the simulated population was generated by random mating, recombination, mutation and sampling of maternal and paternal chromosomes. The simulated population started with 1000 animals with an equal sex ratio, and this structure was kept constant for 1000 generations. Animals were mated by drawing parents randomly from the previous generation, and mating resulted in 1000 offspring (500 males and 500 females) in each generation. A genome containing a single 1-M chromosome was simulated, starting with 2,000 SNP marker loci with positions on the genome determined at random. This density is roughly equivalent to the current SNP chips available for livestock species (e.g. 50 K SNP chip for the 30-M genome in cattle). In the first generation (base population), marker loci were coded as 1 or 2 and allocated at random, so that allele frequencies (p) averaged 0.5. This was comparable to the simulation used in the study of Habier et al. [12]. During the simulation of the 1000 generations, marker alleles were dispersed through the population by random mating, recombinations and mutations. Recombinations between adjacent loci occurred with a probability calculated with Haldane's mapping function, based on the distance between the loci. Mutations occurred for each locus only once during the 1000 generations, where mutations changed the allele state from 1 to 2 or from 2 to 1, with equal probability. Three additional generations were simulated after the first 1000 generations, which were assumed to be genotyped, to analyse genetic diversity over the genome, e.g. similarly as in livestock breeds where only recent generations are genotyped. All SNP markers with a minor allele frequency in generations 1002 and 1003 of <0.02 were discarded from the analysis. Thus, the generated population consisted of 3000 animals (generation 1001, 1002 and 1003) with a known genotype, and 1665 SNP markers were still segregating in these generations.

To determine whether marker density would influence the genetic diversity estimation with the different estimates, a second population was obtained with a lower marker density. This population was based on the first population, by changing only the number of SNP markers from 1665 to 166, by systematically deleting 90% of the SNP markers.

### IBD probabilities

**Figure 1:**
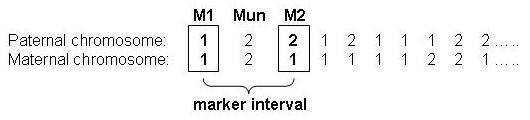
**Definition of marker interval, ungenotyped marker (Mun), and adjacent markers (M1, M2, ...) used for the genetic diversity estimation**. The ungenotyped marker is placed in the middle of the marker interval; genetic diversity was estimated for each marker interval, using the adjacent markers left and right of the interval.

Genetic diversity was estimated for each marker interval on the genome. A marker interval was defined as the interval between two genotyped markers, with one marker lying in between these two markers which was not taken into account for the genetic diversity estimation (ungenotyped marker) (Figure 1). In the next marker interval, this middle ungenotyped marker became the flanking marker of the interval with the adjacent marker being the ungenotyped marker. The genetic diversity estimation was based on IBD probabilities between haplotypes, where a haplotype was defined as a combination of ten consecutive markers, i.e. five markers on either side of the marker interval [6]. Haplotypes were reconstructed from the genotypes using the methods of Windig and Meuwissen [13]. By using IBD probabilities, the chance of markers being similar (AIS) but not IBD is taken into account. This contrasts with heterozygosity, where similar markers are all assumed to originate from the same ancestor (AIS = IBD). Additionally, because haplotypes were used, the recombination history is taken into account to estimate the probability of IBD. For example, a long string of identical markers strongly indicates a recent common ancestor (probability of being IBD must be high), because strings of identical markers from non-recent ancestors are generally broken up by recombination.

IBD probabilities were calculated between the existing haplotypes in the simulated population for each marker interval, by combining linkage disequilibrium and linkage analysis information, where both pedigree and marker information were used. IBD probabilities were first calculated for the first generation of genotyped animals, using the algorithm of Meuwissen and Goddard [6]. In this method, IBD probabilities are calculated for a fictitious locus A in the middle of a marker interval, where information is used from the markers on either side of this locus A. In our case, locus A is positioned at the marker locus in the middle of each marker interval. The probability of A in two haplotypes being IBD or not IBD is estimated by weighing all possible combinations of the markers in the haplotype being IBD or not IBD with recombinations. The IBD probability is calculated back to an arbitrary base population, T generations ago (we used T = 1000). In this calculation, effective population size (we used Ne = 1000 during the 1000 generations) and recombination probabilities based on marker distances are taken into account. As the number of markers with identical alleles increases, the probability that the two fictitious alleles for A are IBD also increases.

After calculating IBD probabilities for the haplotypes in the base generation, the haplotypes of the animals in later generations were added, and the elements in the IBD matrix for those descendant haplotypes were calculated using the algorithm of Fernando and Grossman [9]. In this algorithm, IBD probabilities between offspring are calculated based on the IBD probabilities between the parents and the inheritance of the markers [6]. Whenever the IBD probability of descendant haplotypes with one of their parental haplotypes exceeded 0.95, the descendant haplotype was clustered with this parental haplotype. This was done to avoid excessive numbers of near identical haplotypes resulting in long computation times.

### Genetic diversity based on IBD probabilities

The genetic diversity for all marker intervals on the genome in the simulated population was estimated using haplotype frequencies and IBD probabilities between haplotypes. Haplotype frequencies (frequency of the different haplotype configurations in the population) per marker interval were obtained by:(1)

where ***c***_*i *_is a contribution vector with haplotype frequencies for all haplotypes on marker interval *i*, *N*_*ij *_is the number of haplotypes of type *j *on marker interval *i*, and *N*_*i *_is the total number of haplotypes in the population on marker interval *i*.

Genetic diversity per marker interval was determined by calculating the average haplotype relatedness at each locus [14]:(2)

where *r*_*i *_is the average relatedness for marker interval *i*, and **IBD**_*i *_is the IBD-matrix for marker interval *i*. The genetic diversity for marker interval *i *was calculated as:(3)

This is the predicted probability that the marker in the middle of the interval is not IBD.

### Heterozygosity

Expected heterozygosity [5] was calculated for each marker interval on the genome in the simulated population, using one flanking marker on either side of the interval. Heterozygosity was calculated in two different ways: average heterozygosity of the two adjacent markers around the marker interval (H_exp__AVG), and heterozygosity for the interval treating both markers as a single two-marker haplotype (H_exp__HAP2). For the calculation of H_exp__AVG, first expected heterozygosity was calculated for the markers on the left and right of the interval separately (see Figure 1, markers on the left and right of the interval are in bold):(4)

where *p *and *q *are the allele frequencies for marker *j *in the simulated population. Subsequently, the expected heterozygosity for each marker interval (H_exp__AVG) was calculated by taking the average of the expected heterozygosity for both markers left and right of the marker interval.

H_exp__HAP2 was calculated for the combination of the two markers on the left and right of the interval as a two-marker haplotype (see Figure 1, haplotype is shown with the two markers in bold), where four combinations were possible (11, 12, 21, and 22). H_exp__HAP2 for marker interval *i *was calculated as:(5)

where *p*_*i *_is the frequency of the haplotype with combination *k *at marker interval *i*.

### Comparison GD_IBD and heterozygosity

Comparison between genetic diversity measures GD_IBD, H_exp__AVG and H_exp__HAP2 was done by calculating Pearson's correlations. Correlations were calculated between the genetic diversity measures for each marker interval, but also between the measures averaged over groups of adjacent marker intervals, to investigate whether the correlations would change when the measures were averaged over larger regions of the genome. Therefore, correlations were calculated between GD_IBD, H_exp__AVG and H_exp__HAP2 for 4, 10, 20 and 40 marker intervals together. For example, for 10 marker intervals together, the correlations were calculated with the average measures for interval 1-10, 11-20, 21-30, etc.

### Comparison with true diversity

To evaluate whether one of the approaches better predicts genetic diversity, a true genetic diversity was calculated for the ungenotyped marker lying within each marker interval. This marker was not used to estimate genetic diversity with GD_IBD, H_exp__AVG and H_exp__HAP2, but the adjacent markers were used to predict the diversity in this ungenotyped marker. The true genetic diversity for the ungenotyped marker in the marker interval was determined by calculating the expected heterozygosity (Equation 4). To compare true genetic diversity (H_exp__TRUE) with GD_IBD and heterozygosity (H_exp__AVG and H_exp__HAP2), Pearson's correlations were calculated for each marker interval and for groups of marker intervals (4, 10, 20 and 40). Two correlations were estimated for each comparison: between true genetic diversity of the even markers and their estimated genetic diversity based on the uneven (flanking) markers, and the other way around. This was done because the genotyped marker in one marker interval became the ungenotyped marker in the next marker interval.

## Results

### Simulated population

In the simulated data, 1665 SNP markers were still segregating in generations 1001, 1002 and 1003. Marker distances ranged from 0.00 cM to 0.50 cM, with an average of 0.06 cM. The number of marker haplotypes used for GD_IBD after clustering varied from 1 to 56, with an average of 20.70 haplotypes. The average minor allele frequency over the 1665 SNP markers was 28%, ranging from 2 to 50%. The average linkage disequilibrium (r^2^) between adjacent markers, calculated as the square of the correlation of allele frequencies [15], was 0.26. The simulated population was comparable to real livestock populations. For example, in cattle nowadays ~50,000 SNPs are used for a 30-M genome, which gives an average marker distance of 0.06 cM. On the cattle 50 k SNP chip, for HF dairy cattle the r^2 ^between adjacent markers is between 0.15 and 0.20 for an average marker distance of ~0.06 cM [16,17].

**Figure 2:**
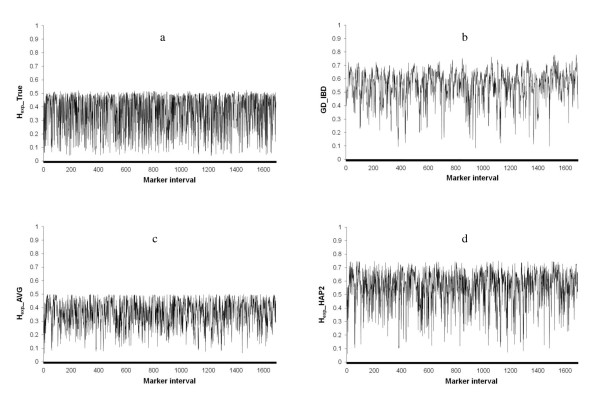
**a, b, c, d - Distribution of the estimated genetic diversity across the simulated genome**. (a) True genetic diversity calculated by expected heterozygosity for the ungenotyped marker loci within the marker interval (H_exp__TRUE); (b) Estimated genetic diversity with IBD probabilities between marker haplotypes (GD_IBD); (c) Estimated genetic diversity with expected heterozygosity as an average for the two flanking markers (H_exp__AVG); (d) Estimated genetic diversity with expected heterozygosity for the two flanking markers as a two marker haplotype (H_exp__HAP2).

**Figure 3:**
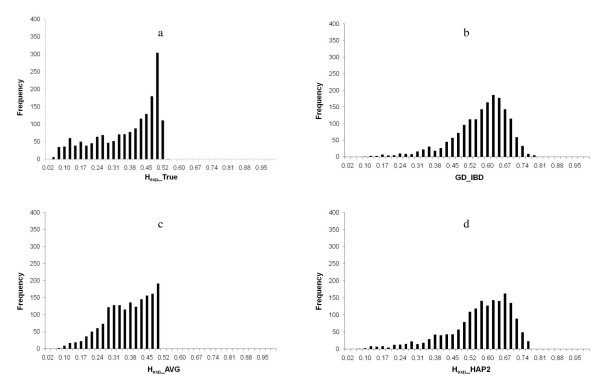
**a, b, c, d - Frequency of the estimated genetic diversity across the simulated genome**. (a) True genetic diversity calculated by expected heterozygosity for the ungenotyped marker loci within the marker interval (H_exp__TRUE); (b) Estimated genetic diversity with IBD probabilities between marker haplotypes (GD_IBD); (c) Estimated genetic diversity with expected heterozygosity as an average for the two flanking markers (H_exp__AVG); (d) Estimated genetic diversity with expected heterozygosity for the two flanking markers as a two marker haplotype (H_exp__HAP2).

The true genetic diversity over the simulated genome, calculated as the expected heterozygosity for the marker locus within each marker interval (H_exp__TRUE), ranged from 0.04 to 0.53 with an average of 0.36 (Figure 2a). A large number of H_exp__TRUE values was found between 0.48 and 0.50 (Figure 3a), which is in accordance with a population in Hardy-Weinberg equilibrium for an allele frequency range 0.4-0.5.

### Genetic diversity estimates

Genetic diversity estimated by IBD probabilities (GD_IBD) varied considerably over the genome, with values ranging from 0.00 to 0.75, with an average of 0.52 (Figures 2b and 3b). Expected heterozygosity calculated for the two adjacent marker loci around each marker interval as an average (H_exp__AVG) resulted in systematically lower values with a smaller range compared to GD_IBD (0.05 to 0.50, average of 0.36) (Figures 2c and 3c). When expected heterozygosity was calculated for flanking markers as a two-marker haplotype (H_exp__HAP2), the level and range of values increased and were more similar to GD_IBD (0.05 to 0.75, average of 0.55) (Figures 2d and 3d). This result was expected, since genetic diversity estimation with H_exp__HAP2 is more similar to GD_IBD because H_exp__HAP2 also uses a haplotype construction, but with only two markers instead of ten. Both heterozygosity estimates fluctuated more over the genome compared to GD_IBD, reflecting a lower correlation between values of adjacent marker intervals for the heterozygosity estimates (H_exp__AVG: r = 0.23; H_exp__HAP2: r = 0.28; GD_IBD: r = 0.64).

### Comparison with true genetic diversity

**Figure 4:**
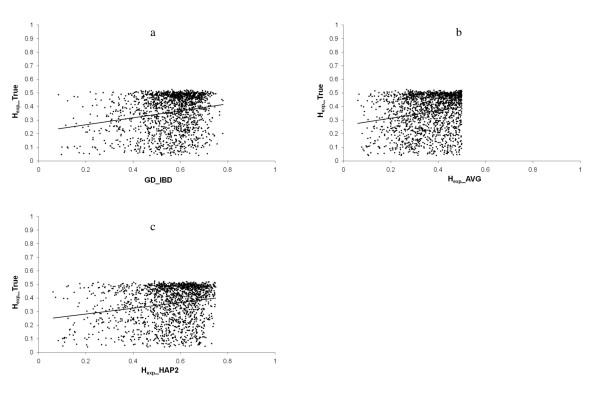
**a, b, c - Relationship between the true genetic diversity (H_exp__TRUE) and estimated genetic diversities**. (a) by IBD probabilities between marker haplotypes (GD_IBD); (b) by expected heterozygosity as an average for the two flanking markers (H_exp__AVG); (c) by expected heterozygosity for the two flanking markers as a two marker haplotype (H_exp__HAP2).

The correlation between H_exp__TRUE and GD_IBD was weak (r = 0.21), and comparable to the correlations between H_exp__TRUE and H_exp__AVG (r = 0.19) and H_exp__HAP2 (r = 0.20) (Table 1 and Figure 4). These results indicate that both GD_IBD and heterozygosity estimates are similar in predicting the genetic diversity for ungenotyped regions of the genome in the current simulated population. The correlation between GD_IBD and H_exp__AVG was 0.46, and was slightly higher between GD_IBD and H_exp__HAP2 (r = 0.49) (Table 1).

**Table 1:** Correlations of true genetic diversity (H_exp__TRUE) with IBD-based diversity (GD_IBD) and heterozygosity (H_exp__AVG and H_exp__HAP2).

MI^a^	True vs. GD_IBD^b^	True vs. H_exp__AVG^b^	True vs. H_exp__HAP2^b^	GD_IBD vs. H_exp__AVG^b^	GD_IBD vs. H_exp__HAP2^b^
1	0.20	0.19	0.20	0.46	0.49
4	0.33	0.27	0.28	0.54	0.58
10	0.46	0.37	0.38	0.64	0.70
20	0.56	0.47	0.50	0.73	0.80
40	0.62	0.61	0.64	0.75	0.82

^a ^The number of marker intervals taken into account to estimate the genetic diversity.

^b ^Correlations were calculated for values per marker interval, and for average values for a group of marker intervals (4, 10, 20 and 40 marker intervals); for the latter, correlations were calculated for the true genetic diversity of even ungenotyped markers with the estimated genetic diversity based on uneven (flanking) markers, and the other way around; the average of both correlations (even and uneven) is presented.

### Comparison with true genetic diversity averaged over marker intervals

When GD_IBD, H_exp__AVG and H_exp__HAP2 were averaged over groups of marker intervals, the correlations between H_exp__TRUE and these estimates increased. They were moderate when estimates were averaged over 40 marker intervals (r = 0.61-0.64, Table 1). Correlations of all three estimates with H_exp__TRUE were comparable to each other. The correlation between GD_IBD and heterozygosity estimates H_exp__AVG and H_exp__HAP2 increased with an increasing number of marker intervals, and in the case of 40 marker intervals equalled 0.75 and 0.82, respectively. This indicates that GD_IBD, H_exp__AVG and H_exp__HAP2 are similar in predicting the genetic diversity for specific regions of the genome in a population with a high marker density.

### Influence of marker density

When genetic diversity over the genome was estimated in a population with a lower marker density, the correlations between the true genetic diversity and GD_IBD, H_exp__AVG and H_exp__HAP2 changed, and turned out to be slightly higher for GD_IBD (Table 2). This result suggests that GD_IBD is a better predictor for genetic diversity when using marker maps with a lower marker density.

**Table 2:** Correlations of true genetic diversity (H_exp__TRUE) with IBD-based diversity (GD_IBD) and heterozygosity (H_exp__AVG and H_exp__HAP2), for a low marker density population (166 SNPs).

MI^a^	True vs. GD_IBD^b^	True vs. H_exp__AVG^b^	True vs. H_exp__HAP2^b^	GD_IBD vs. H_exp__AVG^b^	GD_IBD vs. H_exp__HAP2^b^
1	0.15	0.06	0.04	0.43	0.43
4	0.34	0.18	0.20	0.53	0.53
10	0.51	0.41	0.46	0.79	0.77
20	- ^c^	- ^c^	- ^c^	- ^c^	- ^c^
40	- ^c^	- ^c^	- ^c^	- ^c^	- ^c^

^a ^The number of marker intervals taken into account to estimate the genetic diversity.

^b ^Correlations were calculated for values per marker interval, and for average values for a group of marker intervals (4 and 10 marker intervals); for the latter, correlations were calculated for the true genetic diversity of even ungenotyped markers with estimated genetic diversity based on uneven (flanking) markers, and the other way around; the average of both correlations (even and uneven) is presented.

^c ^There were not enough estimates left over to calculate the correlation.

## Discussion

The aim of this paper was to compare two different estimates of genetic diversity of a region lying in between markers over the genome i.e. IBD-based genetic diversity and heterozygosity. Genetic diversities estimated by IBD probabilities and by heterozygosity of flanking markers were positively correlated. The correlation of GD_IBD and heterozygosity with the true genetic diversity was quite similar for a simulated population with a high marker density, for both specific and large regions over the genome. For a population with a lower marker density, GD_IBD turned out to be a better predictor of genetic diversity.

The assumption that is made for genetic diversity in the ungenotyped marker interval is different for GD_IBD and heterozygosity. With GD_IBD the assumption is that in the base population relatedness was 0, i.e. all markers were not-IBD and "heterozygosity" was 100%. With heterozygosity, no such base population is assumed and the assumption is that heterozygosity in the current generation for genotyped markers is predictive for ungenotyped markers. This explains why the average GD_IBD estimated in this study was higher than the heterozygosity estimates and the true heterozygosity. Heterozygosity based on SNP markers with only two alleles will have, under HWE, a maximum heterozygosity of 50% when the minor allele frequency is 50%, as was simulated in this study. For markers that have an unlimited number of alleles, the true heterozygosity would probably be on average closer to GD_IBD, while for markers with a low diversity the true heterozygosity would be below both GD_IBD and heterozygosity estimates.

When the genotyped marker is actually part of the gene of interest, e.g., when the marker is a known QTL, then heterozygosity at the marker fully determines the additive genetic variance due to the QTL. In that case, additive genetic variance due to the QTL simply equals H_exp_α^2^, α denoting the allele substitution effect of the gene [5]. Hence, when markers coincide with genes of interest, i.e. there are no QTL other than the genotyped markers, there is no need to consider IBD probabilities. However, in most cases, the genes of interest and their QTL will be unknown, and it is unlikely that they coincide precisely with genotyped markers. Consequently, prediction of diversity in the ungenotyped regions between markers is more relevant than the expected diversity at the markers, because most genes of interest will be in the regions between two markers. Such a prediction requires LD between the genotyped markers and the regions in-between markers, similar to the requirements in QTL mapping [18]. Our results show that the IBD-based method and heterozygosity are similar in using LD information in the current simulated data with 1665 SNP markers. However, when a population with a lower marker density was used, GD_IBD became a slightly better predictor of the genetic diversity in the marker interval. In this second population the LD between markers is low due to a larger marker distance, and in that case the IBD-based method was expected to be a better predictor, based on QTL mapping and genomic selection studies. Explaining genetic diversity at a ungenotyped locus is similar to the approaches of QTL mapping and genomic selection, where the objective is to predict genetic variance at one or more unobserved QTL. In those approaches, it has been shown that using an IBD-based method to predict genetic variance at the unobserved QTL is beneficial when the LD between the marker(s) and the QTL is low, while this benefit disappears when the LD increases [10,19].

In our study we ignored the non-segregating SNP markers, as these markers are fixed in the simulated population and show no variation. This can be compared with common practice where base pairs for which no SNP markers are detected are considered uninformative. However, we do not know whether this variation was never there or existed in earlier generations and disappeared. In the latter case, these base pairs indicate a genetic diversity of 0, and should not be ignored. In addition, when non-segregating markers are used in another population, they might show variation and become informative. However, the correlations between the different estimates for genetic diversity as estimated in this paper are unlikely to be influenced by the exclusion of non-segregating markers.

In this study, the estimation of genetic diversity was done for a neutral genome without selection. The correlation between genetic diversity estimates and true genetic diversity was weak, but might increase if adaptive trait variation is taken into account. The availability of dense marker maps has opened up new possibilities to identify reduced or increased levels of variability on specific regions of the genome, associated to functional genes [8]. In case of selection, larger regions with less variation can be found on the genome [20] and a better prediction of the genetic diversity is possible.

How well the two methods predict genetic diversity depends on the variation in diversity between adjacent markers. In contrast to GD_IBD, the heterozygosity estimates assume that diversity is similar for adjacent markers and for instance ignore recombination. When regions of the genome form 'haplotype blocks', adjacent markers have (near) identical diversity. In this case, heterozygosity will better predict the genetic diversity. This was seen when we simulated a population with an effective population size of 100 instead of 1000, and 'haplotype blocks' occurred due to the loss of variation. In this population the correlation between the heterozygosity estimate H_exp__AVG and the true genetic diversity was higher compared to the correlation between GD_IBD and the true genetic diversity (0.97 and 0.90, respectively). However, when a population contains more variation, diversity in between markers can be missed by heterozygosity, as heterozygosity is only based on the variation of the markers itself. In that situation, GD_IBD also takes into account the variation and possible recombination in between markers, and is then expected to be a better estimator of the genetic diversity over the genome. Consequently, as shown in this study the method of choice will also depend on the marker density [10,19], with high marker densities (i.e. > 50 markers per cM) heterozygosity is likely to perform better, with lower marker densities (i.e. <10 markers per cM) GD_IBD is likely to perform better.

## Conclusions

In conclusion, the IBD-based method and heterozygosity used to estimate genetic diversity of ungenotyped regions of the genome (i.e. between markers) give similar results for a simulated population with a high marker density. However, for a population with a lower marker density, the IBD-based method gives a better prediction, since variation and recombination between markers are missed with heterozygosity. IBD-based methods can provide more insight in the genetic diversity of specific regions of the genome, and subsequently contribute to select more accurately the animals to be conserved, for example, to construct a gene bank.

## Competing interests

The authors declare that they have no competing interests.

## Authors' contributions

KAE developed part of the programs used for analysis, carried out the simulations and analyses, and wrote most of the paper. MPLC developed most of the programs used for the simulations and analysis, and supervised and advised KAE. PB contributed to part of the discussion and supervised and advised KAE. JJW conceived the study, participated in its design and coordination, mentored and advised KAE daily, and contributed parts of the paper. All authors took part in useful discussions, and provided useful advice on the analyses and the first draft of the paper. All authors read and approved the final manuscript.
